# Aromatic Amino Acid Activation of Signaling Pathways in Bone Marrow Mesenchymal Stem Cells Depends on Oxygen Tension

**DOI:** 10.1371/journal.pone.0091108

**Published:** 2014-04-11

**Authors:** Mona El Refaey, Qing Zhong, William D. Hill, Xing-Ming Shi, Mark W. Hamrick, Lakiea Bailey, Maribeth Johnson, Jianrui Xu, Wendy B. Bollag, Norman Chutkan, Carlos M. Isales

**Affiliations:** 1 Institute for Regenerative and Reparative Medicine, Georgia Regents University, Augusta, Georgia, United States of America; 2 Department of Medicine, Medical College of Georgia, Georgia Regents University, Augusta, Georgia, United States of America; 3 Department of Orthopaedic Surgery, Medical College of Georgia, Georgia Regents University, Augusta, Georgia, United States of America; 4 Department of Cellular Biology and Anatomy, Medical College of Georgia, Georgia Regents University, Augusta, Georgia, United States of America; 5 Department of Pathology, Medical College of Georgia, Georgia Regents University, Augusta, Georgia, United States of America; 6 Department of Physiology, Medical College of Georgia, Georgia Regents University, Augusta, Georgia, United States of America; 7 Department of Biostatistics, Medical College of Georgia, Georgia Regents University, Augusta, Georgia, United States of America; 8 Charlie Norwood VA Medical Center, Augusta, Georgia, United States of America; Georgia Regents University, United States of America

## Abstract

The physiologic oxygen pressures inside the bone marrow environment are much lower than what is present in the peripheral circulation, ranging from 1–7%, compared to values as high as 10–13% in the arteries, lungs and liver. Thus, experiments done with bone marrow mesenchymal stem cells (BMMSCs) using standard culture conditions may not accurately reflect the true hypoxic bone marrow microenvironment. However, since aging is associated with an increased generation of reactive oxygen species, experiments done under 21%O_2_ conditions may actually more closely resemble that of the aging bone marrow environment. Aromatic amino acids are known to be natural anti-oxidants. We have previously reported that aromatic amino acids are potent agonists for stimulating increases in intracellular calcium and phospho-c-Raf and in promoting BMMSC differentiation down the osteogenic pathway. Our previous experiments were performed under normoxic conditions. Thus, we next decided to compare a normoxic (21% O_2_) vs. a hypoxic environment (3% O_2_) alone or after treatment with aromatic amino acids. Reverse-phase protein arrays showed that 3% O_2_ itself up-regulated proliferative pathways. Aromatic amino acids had no additional effect on signaling pathways under these conditions. However, under 21%O_2_ conditions, aromatic amino acids could now significantly increase these proliferative pathways over this “normoxic” baseline. Pharmacologic studies are consistent with the aromatic amino acids activating the extracellular calcium-sensing receptor. The effects of aromatic amino acids on BMMSC function in the 21% O2 environment is consistent with a potential role for these amino acids in an aging environment as functional anti oxidants.

## Introduction

Little is known about direct nutrient effects on bone marrow mesenchymal stem cells (BMMSCs). However, previous findings have suggested that nutritional signaling pathways can activate BMMSCs and undergo age-dependent suppression to result in bone loss [1], [2]. Here we focused on amino acids as epidemiological data supported an association between protein intake and anabolic effects on bone [3], [4], as well as between low protein intake and a greater incidence of hip fracture in the elderly.

Since BMMSCs differentiate into either bone-forming/osteoblastic phenotypes or adipocytes, they would appear to be natural targets for nutrient activation. Recent research by others [5] shows that L-type amino acids modulate several members of the class 3 G-protein-coupled receptor superfamily, including the extracellular calcium- sensing receptor (CaSR), which is modulated by aromatic, aliphatic and polar amino acids, and member 6A of the G-protein-coupled receptor family C (GPRC6A). Cationic amino acids (e.g. ornithine and arginine) are the most potent amino acid activators of GPRC6A and aromatic amino acids are the most potent amino acid activators of the CaSR. The binding of Ca^2+^ ion to the CaSR activates multiple signaling pathways that regulate proliferation, cell growth, cell cycle and differentiation. Interactions between the CaSR and amino acid metabolism are suggested by recent data showing marked elevation of parathyroid hormone (PTH) levels during protein restriction [6] and long-standing data showing that high protein intake promotes urinary calcium excretion [7], [8].

The decline of tissue regenerative potential with age can be reversed through the modulation of systemic factors this suggests that tissue-specific stem and progenitor cells retain much of their intrinsic proliferative potential even when old and that the age related changes in the systemic environment and niche in which progenitor cells reside preclude full activation of these cells for productive tissue regeneration [9]. Human MSCs are primarily sequestered within the bone marrow, where they exist in a symbiotic relationship with hematopoietic stem cells (HSCs) [10]. The close interactions between these two types of stem cells strongly suggest that they reside in similar niches within the bone marrow and are affected by the same environmental cues. HSCs reside in severely hypoxic regions of the bone marrow and maintaining HSCs under hypoxic conditions contributes significantly to their proliferation, expansion and self-renewal capabilities [11], [12]. Therefore, we sought to determine the ability of amino acids to affect BMMSCs under normoxic versus hypoxic conditions.

## Materials and Methods

### Isolation and Culture of BMMSCs

All experiments were approved by the Institutional Animal Care and Use Committee at Georgia Regents University (Protocol #: BR09-11-265; Augusta, GA). The mice were housed in AAALAC accredited facilities under the supervision of a veterinarian. Georgia Regents University complies with the NIH policy on animal welfare, the Animal Welfare Act, and all other applicable federal, state and local laws. All available measures were taken to minimize pain and suffering. Male C57BL/6 mice were purchased from the National Institute on Aging (Bethesda, MD, USA) aged rodent colony. BMMSCs were isolated from 18-month-old male C57BL/6 mice at the Georgia Regents University Stem Cell Core Facility. In brief, six mice were euthanized by CO_2_ overdose followed by thoracotomy. Whole bone marrow aspirates were flushed from femora and tibiae and BMMSCs were isolated by negative immunodepletion using magnetic micro-beads conjugated to anti-mouse CD11b (cat#558013), CD45R/B220 (cat#551513) (BD Biosciences Pharmingen, San Diego, CA, USA), CD11c, and plasmacytoid dendritic cell antigen (PDCA)-1 (cat#130-092-465) (Miltenyi Biotec, Bergisch Gladbach, Germany) followed by positive immuno-selection using anti-stem cell antigen (Sca)-1 micro-beads (cat#130-092-529) (Miltenyi Biotec) according to the manufacturer's recommendations. The calcium sensing receptor antagonist NPS-2143 was purchased from Sigma-Aldrich, St. Louis, MO. BMMSCs were grown in Dulbecco's Modified Eagle Medium (cat#10-013) (DMEM; Cellgro, Mediatech, Manassas, VA, USA) supplemented with 10% heat-inactivated fetal bovine serum (cat#S11150) (FBS; Atlanta Biologicals, Lawrenceville, GA, USA) and 1% penicillin-streptomycin (cat#SV30010) (Hyclone Laboratories, Inc.) [2], [13].

### Western Blotting

Whole cell lysates of treated BMMSCs were prepared in complete Lysis-M EDTA-free buffer containing protease inhibitors (cat#04719964001) (Roche Diagnostics, Indianapolis, IN, USA). Equal amounts (50 µg) of protein lysates were subjected to SDS-PAGE and transferred to 0.45 µm PVDF membranes (cat#IPFL00010) (Millipore, Billerica, MA, USA). Membranes were blocked with blocking buffer (cat#927-40000) (Licor Biosciences). ERK (extracellular signal-regulated kinase) signals were detected using specific primary antibodies to pERK (cat#4370) (dilution1∶400) and ERK (cat#4696) (dilution 1∶1000) (Cell Signaling Technology, Danvers, MA, USA followed by secondary antibodies, anti-rabbit IgG goat antibody IRDye 800 Conjugated (cat # 611-132-122; Rockland Immunochemicals Inc. Gilbertsville, PA) and alexa Fluor 680 Goat Anti-Mouse IgG (H+L), highly cross-adsorbed (cat#A21058, Invitrogen). Bound antibodies were visualized using a Licor scanner and quantified using Licor image analysis software.

### Preparation of cell lysate for Reverse-Phase Protein Array

Two sets of 18-month-old BMMSCs were seeded under two different conditions. One set was grown under normoxic conditions (21% O_2_/5% CO_2_) and the other set under low O_2_ tension (3% O_2_/5% CO_2_; physiologic hypoxia).

Different treatment groups (when cells were 80% confluent) consisted not only of normoxia versus hypoxia but also comparing the effects of aromatic amino acids: phenylalanine (Phe), tyrosine (Tyr), tryptophan (Trp); a branched chain amino acid, valine (Val); and threonine (Thr) using a 100 µM concentration under both oxygen tensions. The cells were treated in serum-free media for 3 h. The cells were washed twice with PBS and lysis buffer was added to the plates. Lysis Buffer consisted of 1% Triton X-100, 50 mM HEPES, pH = 7.4, 150 mM NaCl, 1.5 mM MgCl_2_, 1 mM EGTA, 100 mM NaF, 10 mM Na pyrophosphate, 1 mM Na_3_VO_4_, 10% glycerol containing freshly added protease and phosphatase inhibitors (cat # 05056489001 and 04906837001) (Roche Applied Science). The plates were incubated on ice (leveled) for 20 minutes with occasional shaking every 5 minutes. The cells were scraped off the plates and the cell lysate was collected into micro-centrifuge tubes. The cell lysate was then centrifuged at 14,000 rpm (maximum speed) for 10 minutes at 4°C. The supernatant was then carefully collected and the pellet was discarded. The cellular protein concentration was determined by Bradford reaction and protein concentration was adjusted to 1–1.5 mg/ml. The cell lysate was mixed with 4X SDS sample buffer without bromophenol blue (3 parts of cell lysate plus one part of 4X SDS sample buffer). The samples were boiled for 5 min to be ready for RPPA processing.

### Reverse-phase protein array

RPPA cell lysates were two-fold serially diluted for 5 dilutions (from undiluted to 1∶16 dilution) and arrayed on a nitrocellulose-coated slide in an 11×11 format. Samples were probed with antibodies by catalyzed signal amplification (CSA) and visualized by a DAB colorimetric reaction. Samples were probed for 172 antibodies, which were analyzed on ArrayPro then by supercurve R x64 2.15.1. There were 22 sets of replicated antibodies among 172 antibodies. QC tests were performed for each antibody staining (slide) with a QC score above 0.8 indicating good antibody staining. In the case of antibodies with replicates the one with the highest QC Score was used. Antibodies were classified according to their validation status into valid, use with caution and under evaluation; only validated ones that showed a correlation between RPPA and western blot above 0.7 were selected for discussion in our Results section. Slides were scanned on a flatbed scanner to produce a 16-bit tiff image and spots from tiff images were identified and the density was quantified by Micro-Vigene. Relative protein levels for each sample were determined by interpolation of each dilution curve from the “standard curve” (super-curve) of the slide (antibody). Super-curves are constructed by a script in R written by Bioinformatics. These values (given as Log2 values) are defined as Super-curve Log2 (Raw) value. Normalized linear values were used in the statistical analysis [14].

### Statistical Analysis

Experiments were performed at least three independent times. For RPPA data, all data are expressed as means ± SEM. Two-sided one-sample t-tests were performed for the hypothesis that the ratio not equal to 100% (ratio is treatment/control). For western blot analyses, data are expressed as mean ± SEM. Upper tail one-sample t-tests were performed for the hypothesis that the expression relative to control was greater than 100. A log transformation was used to stabilize the variance. Null hypotheses were rejected at the 0.05 level. No multiple testing adjustments were made. Data were analyzed using SAS© 9.3 (SAS Institute, Inc., Cary, NC).

## Results

### Normoxia versus hypoxia

**Figure 1:**
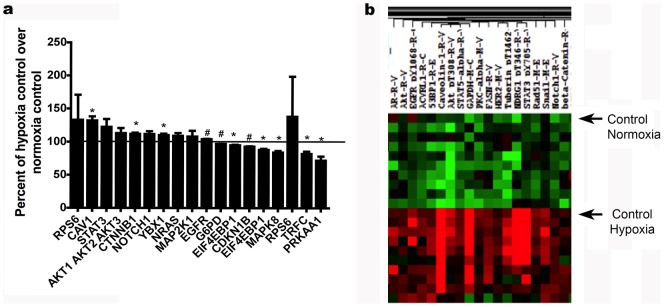
Hypoxia itself modulates BMMSC signaling pathways. Reverse phase protein arrays were performed under either normoxic or hypoxic conditions. **Panel A**: RPPA graph of two sets of untreated BMMSCs grown under normoxic conditions or low O_2_ tension. When cells were 80% confluent they were switched to serum-free media for 3 h. Then cells were washed twice with PBS and lysis buffer was added to the plates. Results are expressed as means ±SEM for three independent experiments. *p≤0.05 and # *p*≤0.01. Percent change of untreated BMMSCs in hypoxia was determined as percent change of untreated BMMSCs in normoxia (control = 100%). RPS6: Ribosomal protein s6 (S6_pS240_S244-R-V). CAV1: Caveolin 1 (Caveolin-1-R-V). STAT3: Signal transducer and activator of transcription 3 (STAT3_pY705-R-V). AKT1 AKT2 AKT3: protein kinase B (Akt_pT308-R-V). CTNNB1: β-catenin (beta-Catenin-R-V). NOTCH1: Notch homolog 1 (Notch1-R-V). YBX1: The Y-box-binding Protein (YB-1-R-V). NRAS: Neuroblastoma RAS viral (v-ras) oncogene (N-Ras-M-V). MAP2K1: Mitogen activated protein kinase (MEK1-R-V). EGFR: Epidermal growth factor receptor (EGFR_pY1173-R-V). G6PD: Glucose-6 phosphate dehydrogenase (G6PD-M-V). EIF4EBP1: Eukaryotic initiating factor 4 (4E-BP1-R-V). CDKN1B: Cyclin dependent kinase inhibitor (p27_pT198-R-V). EIF4EBP1: Eukaryotic initiating factor 4 (4E-BP1_pS65-R-V). MAPK8: c-Jun N-terminal kinases (JNK_pT183_pT185-R-V). TRFC: Total rosette forming cells CD71 marker (TRFC-R-V). PRKAA1: 5'-AMP-activated protein kinase catalytic subunit alpha-1 (AMPK_pT172-R-V). **Panel B**: RPPA heatmap showing up-regulation of Akt (more than one antibody), CAV1, STAT3 and NOTCH1 in untreated BMMSCs in hypoxia vs. untreated BMMSCs in normoxia (control).

To investigate the role of oxygen tension in activating different proliferation pathways, we cultured BMMSCs either in standard culture conditions (21% O_2_/5% CO_2_) or under low O_2_ tension (3% O_2_/5% CO_2_; physiologic hypoxia). In this experiment, hypoxia was considered as a treatment and normoxia was considered the control. The RPPA data with hypoxia showed an up-regulation in the protein kinase B (Akt pathway), which regulates cell proliferation, caveolin 1(CAV1), which among other roles is the initiating step in coupling integrins to the Ras-ERK pathway and promoting cell cycle progression, and ribosomal protein S6 (RPS6), which regulates not only translation and protein synthesis but also cell growth and proliferation. Signal transducer and activator of transcription 3 (STAT3), which has anti-apoptotic and proliferative effects, and the notch 1 (NOTCH1) pathway, which also has a proliferative effect, were also up-regulated. However, AMP-activated protein kinase (AMPK), which is activated in response to environmental stresses, and c-Jun N-terminal protein Kinase (JNK) were down regulated (i.e. decreased activating phosphorylation) under hypoxia as shown in figure 1.

### Effects of aromatic amino acids on BMMSCs proliferation pathways

**Figure 2:**
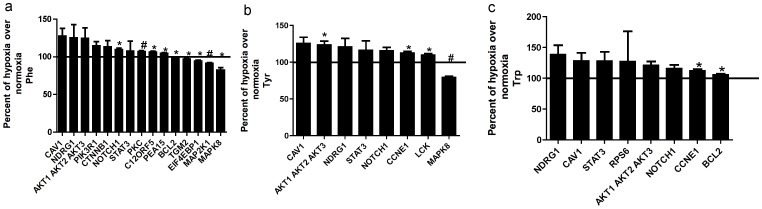
Aromatic amino acid stimulated effects on signaling pathways under normoxic vs hypoxic conditions. Reverse-phase protein array graphs of two sets of BMMSCs grown under normoxic conditions (21% O_2_/5% CO_2_) and under low O_2_ tension (3% O_2_/5% CO_2_; physiologic hypoxia). When cells were 80% confluent they were treated with Phe, Tyr or Trp (100 µM) in serum-free media for 3 h. The cells were washed twice with PBS and lysis buffer was added to the plates. a-Phe, b-Tyr and c-Trp. Results are expressed as means ± SEM for three independent experiments. *p≤0.05 and # *p*≤0.01. Percent change of hypoxia over control for Phe was determined as percent change of BMMSCs treated with Phe under hypoxic conditions (3% O_2_%) vs. BMMSCs treated with Phe under normoxic conditions (21% O_2_) (control = 100%). Both sets of BMMSCs were treated with Phe and grown under different oxygen levels. Same for Tyr and Trp pannels. RPS6: Ribosomal protein s6 (S6_pS240_S244-R-V). CAV1: Caveolin 1 (Caveolin-1-R-V). NDRG1: N-myc downregulated gene (NDRG1_pT346-R-V). AKT1 AKT2 AKT3: protein kinase B (Akt_pT308-R-V). STAT3: Signal transducer and activator of transcription 3 (STAT3_pY705-R-V). CTNNB1: β-catenin (beta-Catenin-R-V). NOTCH1: Notch homolog 1 (Notch1-R-V). MAP2K1: Mitogen activated protein kinase (MEK1-R-V). EIF4EBP1: Eukaryotic initiating factor 4 (4E-BP1_pT37_T46-R-V). MAPK8: c-Jun N-terminal kinases (JNK_pT183_pT185-R-V). LCK: Lymphocyte-specific protein tyrosine kinase (Lck-R-V). CCNE1:Cyclin E1 (Cyclin_E1-M-V). BCl2: B-cell lymphoma 2 (Bcl-2-M-V). PIK3R1: Phosphatidylinositol 3-kinase regulatory subunit alpha (PI3K-p85-R-V). PKC: Protein kinase C (PKC-pan_BetaII_pS660-R-V). C12ORF5: TP53-inducible glycolysis and apoptosis regulator (TIGAR-R-V). PEA15: Phosphoprotein Enriched in Astrocytes 15 (PEA15_pS116-R-V). TGM2: transglutaminase2, C polypeptide, protein-glutamine-gamma-glutamyltransferase(Transglutaminase-M-V).

We later wanted to study the effect of aromatic amino acids (Tyr, Phe and Trp) on BMMSC signaling pathways, since aromatic amino acids are the most potent amino acid activators of the calcium sensing receptor. We decided to compare the effects of all three aromatic amino acids on signaling pathways under normoxic and hypoxic conditions. In these experiments, BMMSCs cultured under normoxic conditions and treated with aromatic amino acids were used as control compared to BMMSCs treated with aromatic amino acids under hypoxic conditions. Our data showed that under hypoxia Phe, Tyr and Trp up-regulated the activating phosphorylation of Akt, CAV1, STAT3 and NOTCH1 (same effect as untreated cells under hypoxia in figure 1), all pathways that play an important role in cell growth and proliferation. All three aromatics also up-regulated N-myc downregulated gene (NDRG1), which is a stress-response protein involved in cell growth and differentiation and is necessary for p53/TP53-mediated caspase activation and apoptosis. Under hypoxic conditions Phe and Tyr down-regulated mitogen-activated protein kinase 8 (MAPK8) and c-Jun N-terminal kinase (JNK) which regulates cell proliferation, differentiation and apoptosis. Tyr and Trp up-regulated cyclin E1, an important marker for cell cycle progression. The RPPA also showed that Trp up-regulated RPS6, as shown in figure 2.

### Effects of polar and branched-chain amino acids on BMMSC proliferation

**Figure 3:**
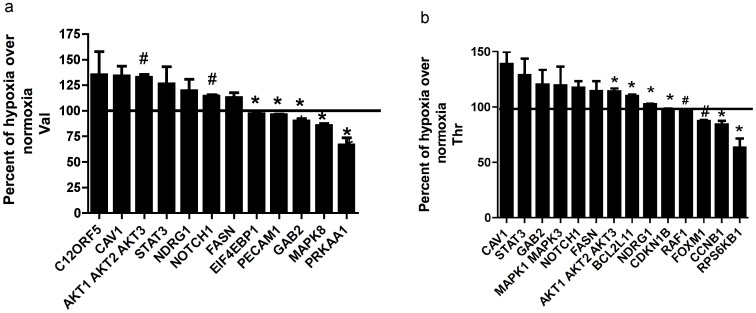
Impact of normoxia on polar or branched chain amino acid stimulated BMMSCs. Reverse-phase protein array graphs of two sets of BMMSCs grown under normoxic conditions (21% O_2_/5% CO_2_) and under low O_2_ tension (3% O_2_/5% CO_2_; physiologic hypoxia). When cells were 80% confluent,The cells were treated with Val or Thr (100 µM) in serum-free media for 3 h. The cells were washed twice with PBS and lysis buffer was added to the plates. a-Val and b-Thr. Results are expressed as means ± SEM for three independent experiments. *p≤0.05 and # *p*≤0.01. Percent change of hypoxia over control for Val was determined as percent change of BMMSCs treated with Val under hypoxic conditions (3% O_2_%) vs. BMMSCs treated with Val under normoxic conditions (21% O_2_) (control = 100%). Both sets of BMMSCs were treated with Val and grown under different oxygen levels. Same for Thr panel. CAV1: Caveolin 1 (Caveolin-1-R-V). NDRG1: N-myc downregulated gene (NDRG1_pT346-R-V). AKT1 AKT2 AKT3: protein kinase B (Akt_pT308-R-V). STAT3: Signal transducer and activator of transcription 3 (STAT3_pY705-R-V). NOTCH1: Notch homolog 1 (Notch1-R-V). FASN: Fatty acid synthase (FASN-R-V). PECAM1: Platelet endothelial cell adhesion molecule (CD31-M-V). GAB2: GRB2-associated-binding protein 2 (Gab2-R-V). PRKAA1: 5'-AMP-activated protein kinase catalytic subunit alpha-1 (AMPK_pT172-R-V). MAPK1 MAPK3: Mitogen-activated protein kinase (MAPK_pT202_Y204-R-V). CDKN1B: Cyclin-dependent kinase inhibitor 1B (p27-R-V). RAF1: Proto-oncogene serine/threonine-protein kinase (C-Raf-R-V). FOXM1: Fork-head box protein M1 (FoxM1-R-V). CCNB1: Cyclin B1 (Cyclin_B1-R-V). RPS6KB1: Ribosomal protein S6 kinase beta-1 (p70S6K_pT389-R-V). EIF4EBP1: Eukaryotic initiating factor 4 (4E-BP1-R-V). MAPK8: c-Jun N-terminal kinases (JNK_pT183_pT185-R-V). BCl2L11: Bcl-2-like protein 11(Bim-R-V). C12ORF5: TP53-inducible glycolysis and apoptosis regulator (TIGAR-R-V).

To further study the effects of amino acids on proliferation signaling pathways, we treated the cells with Val which is similar to leucine and isoleucine in being a branched-chain amino acid and Thr similar to serine in being polar. We treated BMMSCs with these amino acids in normoxia and hypoxia and treated cells in normoxia were used as control. Arrays showed that Val and Thr up-regulated Akt, notch 1, STAT3 and cav1 under hypoxia which are all important for cell growth and proliferation. Val up-regulated TP53-inducible glycolysis and apoptosis regulator (TIGAR) which protects cells against reactive oxygen species and against apoptosis induced by p53/TP53, and NDRG1, which is also a stress responsive protein in hypoxic conditions. Thr up-regulated GRB2-associated-binding protein 2 (GAB2), which mainly activates the ERK/MAPK pathway (MAPK1 MAPK3) that regulates cell proliferation. Data also showed that Val down-regulated AMPK, activated in response to environmental stresses, and JNK that regulates cell proliferation. Thr down-regulated Fork-head box protein M1 (FOXM1) which is activated to protect against reactive oxygen species, as well as cyclin B1 (CCNB1) and ribosomal protein S6 kinase beta-1 (RPS6KB1), which also plays a role in response to oxidative stress as shown in figure 3.

### Effects of amino acids versus control under normoxic conditions

**Figure 4:**
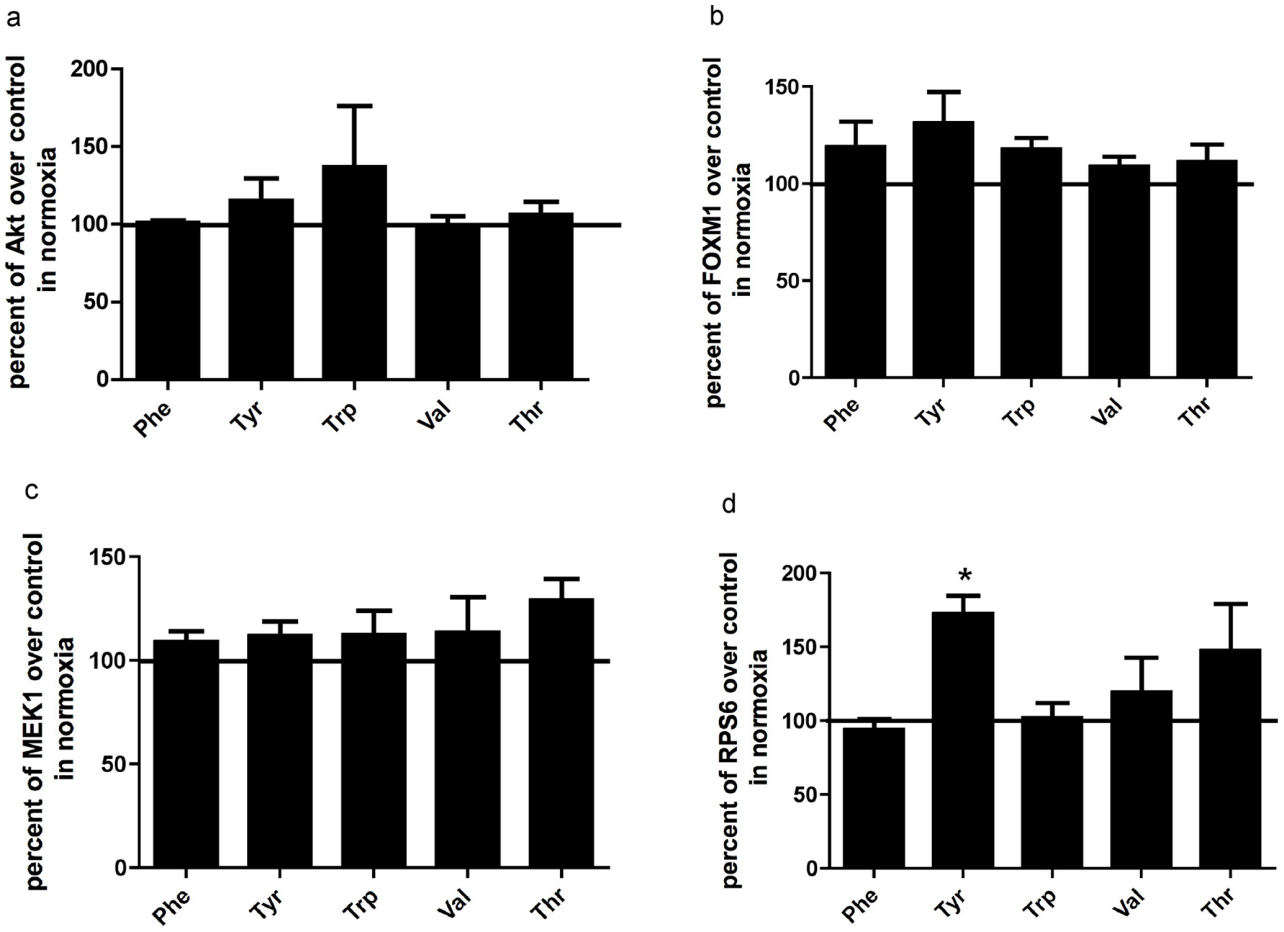
Effects of aromatic amino acids on signaling pathways under normoxia. Reverse-phase protein array graphs of BMMSCs grown under normoxic conditions (21% O_2_/5% CO_2_). When cells were 80% confluent, they were treated with either Phe, Tyr, Trp, Val or Thr (100 µM) in serum-free media for 3 h and compared to the untreated control (100%). **a-**Akt pathway **b-**FOXM1 **c-**MAP2K1 and **d-** RPS6. The cells were washed twice with PBS and lysis buffer was added to the plates. Results are expressed as means ± SEM for three independent experiments. *p≤0.05 and # *p*≤0.01. AKT1 AKT2 AKT3: protein kinase B (Akt_pT308-R-V). FOXM1: Fork-head box protein M1 (FoxM1-R-V). MAP2K1: Mitogen activated protein kinase (MEK1-R-V), RPS6:Ribosomal protein s6 (S6_pS240_S244-R-V).

Under normoxic conditions, Tyr and Trp induced an up-regulation of the Akt pathway. Tyr and Trp elicited an up-regulation of FOXM1, which, as mentioned previously, is activated to protect against reactive oxygen species. Untreated BMMSCs under normoxia were used as control. RPPA showed that Tyr up-regulated RPS6, which plays a major role in translation and protein synthesis. Results also showed that Tyr andThr up-regulated RPS6 that controls protein synthesis and translation (figure 4).

### Effects of aromatic amino acids on MAPK pathway

**Figure 5:**
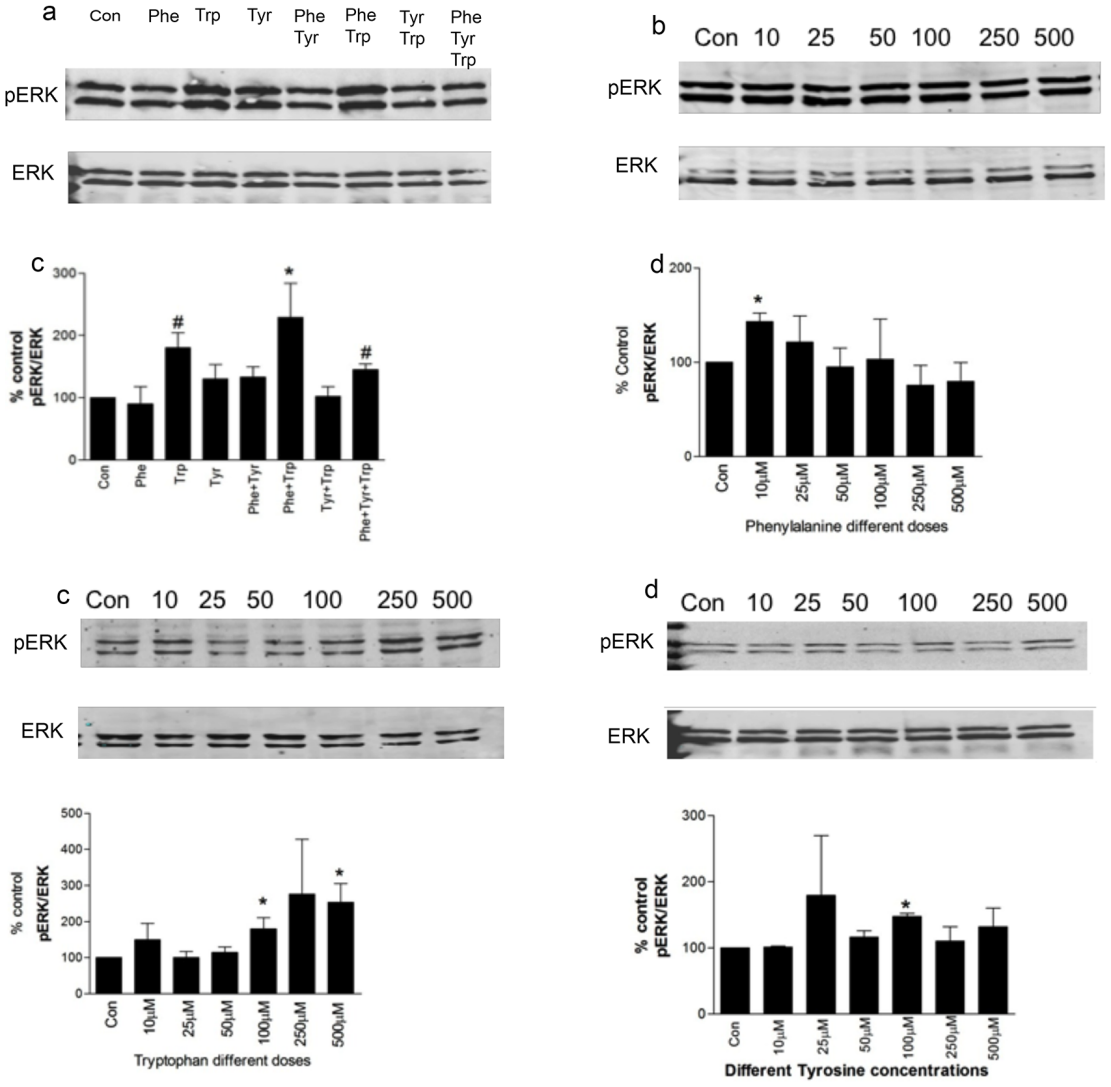
Effect of aromatic amino acids on the MAPK pathways. Western blot graphs of BMMSCs grown under normoxic conditions (21% O_2_/5% CO_2_). When cells were 80% confluent, they were treated with either Phe, Tyr or Trp alone and in combinations (100 µM) in serum-free media and compared to the untreated BMMSCs (control = 100%). The cells were washed twice with PBS and lysis buffer was added to the plates. Results are expressed as means ± SEM for at least three independent experiments. *p≤0.05 and # *p*≤0.01. **a-**Different aromatic amino acids singly or in combinations as indicated (100 µM). **b-**Phe different doses (0–500 µM). **c-**Trp different doses (0–500 µM). **d-**Tyr different doses (0–500 µM).

Next, the effects of aromatic amino acids on the MAPK pathway and specifically ERK phosphorylation, with the amino acids applied singly and in combination were examined using the western blot technique in normoxic conditions. We also studied the effects of different doses of aromatic amino acids on ERK phosphorylation. Trp alone and in combination showed a two-fold increase in ERK phosphorylation when compared to the control (untreated BMMSCs). Phe showed a biphasic effect on ERK phosphorylation so that lower doses had a greater effect on ERK phosphorylation than higher doses. Trp showed a dose dependent effect in which higher doses produced an increase in ERK phosphorylation. Tyr showed no dose-dependent effect as shown in figure 5.

**Figure 6:**
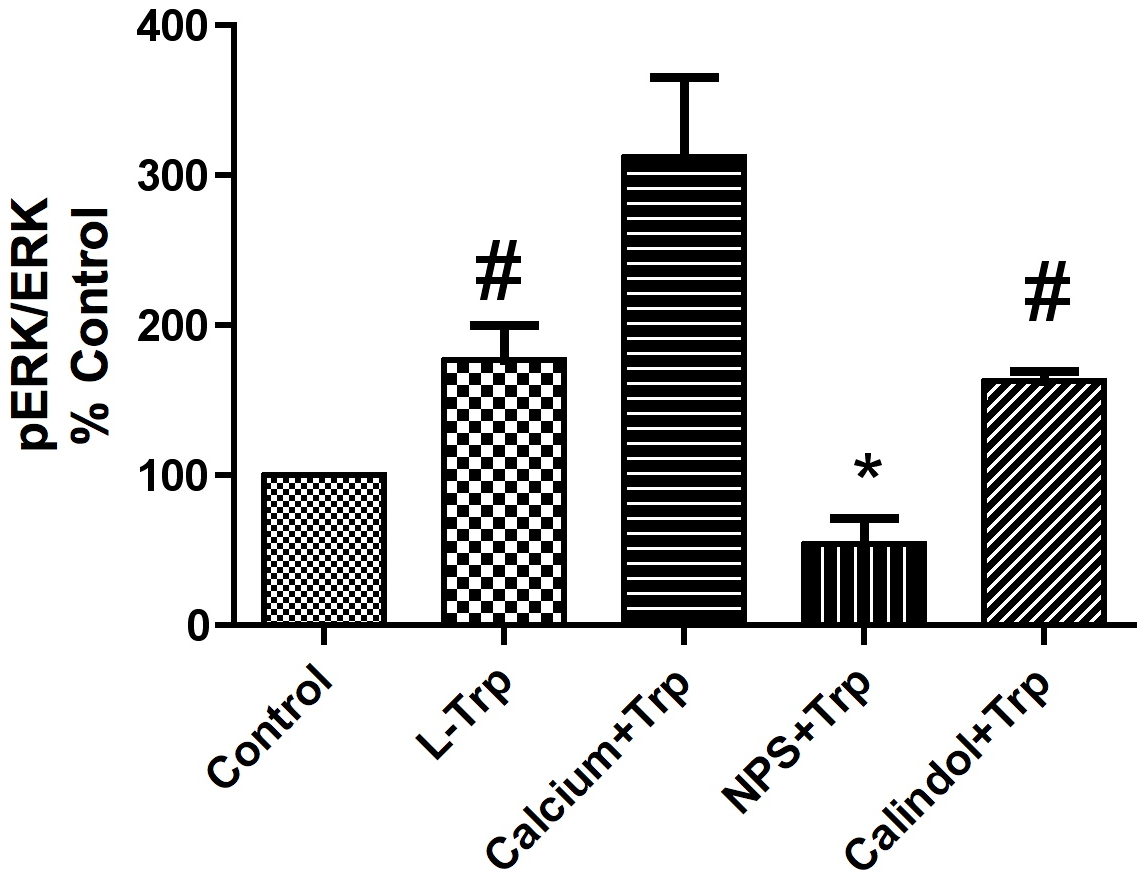
Modulatory effects of calcium on Tryptophan stimulated ERK phosphorylation. Erk (phospho Erk/Total Erk) assesed by Western blots of BMMSCs grown under normoxic conditions (21% O_2_/5% CO_2_). When cells were 80% confluent, they were treated with either Trp alone (100 µM) or in combination with either additional extracellular calcium (baseline calcium: 1.2 mM increased to 1.8 mM) or a calcium receptor antagonist (NPS 10 nM) in serum-free media. The cells were washed twice with PBS and lysis buffer was added to the plates. Results are expressed as means ± SEM for at least three independent experiments. *p≤0.05 and # *p*≤0.01.

Effects of calcium and a calcium receptor antagonist on Tryptophan stimulated ERK phosphorylation:

In an effort to better define the mechanism of aromatic amino acid action on BMMSC the effects of increasing extracellular calcium and the CaSR antagonist NPS 2143 were examined next. Tryptophan (100 µM) itself significantly increased ERK phosphorylation. Increasing the extracellular calcium concentration in the medium from 1.2 to 1.8 mM further significantly increased ERK phosphorylation (Figure 6). In contrast when the calcium receptor antagonist NPS 2143 (10 nM) was added to the BMMSC, it significantly inhibited Tryptophan stimulated ERK phosphorylation.

## Discussion

The present study had two aims: (1) to evaluate direct amino acid effects on cell signaling pathways; and (2) to study the impact of oxygen tension on the nutrient signaling pathways activated in BMMSCs. Our group previously demonstrated an activation in BMMSCs of nutritional signaling pathways in BMSCs that undergo age-dependent suppression to result in bone loss [1],[2]. Moreover, we have focused on nutrients (amino acids) for this study because data from our laboratory demonstrated that they are critical for BMMSC survival as we had made the observation that if BMMSCs were grown in Medium 199 instead of DMEM, the cells died within 3 days (data not shown). We examined the formulae for both media and found that the main difference between DMEM and Medium 199 was their amino acid content. Our pharmacologic data using increasing extracellular calcium and the CaSR antagonist NPS 2143 are consistent with aromatic amino acids binding to the CaSR and activating proliferative pathways. These results of amino acid activating the CaSR would be consistent with those reported by others [15], [16]. The C57BL/6 mouse model was used due to results consistent with human clinical data, and because it is a model previously characterized by our group [17]. We wanted to determine the ability of amino acids to affect BMMSCs under normoxic versus hypoxic conditions, as human MSCs are primarily sequestered within the bone marrow, where they exist in a symbiotic relationship with hematopoietic stem cells (HSCs) [10] that reside in severely hypoxic regions.

Our data showed that culturing BMMSCs under low oxygen tension (3% O_2_/5% CO_2_; physiologic hypoxia) up-regulated signaling pathways such as Akt, STAT3, CAV1 and NOTCH1 regardless of treatment, as these pathways were up-regulated in the untreated control as well as the amino acid-treated cells. It was previously reported that genetic modification of BMMSCs with the pro-survival gene Akt1 (Akt-BMSCs) increases the post-transplantation viability of these cells and enhances their therapeutic efficacy [18]. We provide here evidence that hypoxic conditioning of BMMCS increases the Akt signaling pathway that regulates cell survival and proliferation. Our array also showed an up-regulation of STAT3 which correlates with previous work done by others[19], which showed that hypoxia induced more activation of STAT3 in correlation with higher VEGF production in hypoxic BMMSCs. These data suggest that oxygen tension has an impact on the signaling pathways activated in BMMSCs and that results under “hypoxic” conditions more closely resemble the normal physiologic conditions these cells are exposed to in their niches. However, oxidative stress is increased with aging, suggesting that the pathophysiology of aging may correlate with conditions experienced by the BMMSCs under normoxic conditions.

Under normoxic conditions, our data showed that Tyr up-regulated FOXM1, which is activated in response to reactive oxygen species. Previous research on FOXM1 showed that elevated levels of FoxM1, in turn, down-regulate ROS levels by stimulating expression of ROS scavenger genes, such as manganese superoxide dismutase (MnSOD) and catalase. FoxM1 depletion sensitizes cells to control oxidative stress resulting in premature senescence and apoptosis [20]. Trp under normoxia also induced an up-regulation of the pro-survival Akt protein. Normoxic data also showed that Tyr up-regulated RPS6, which plays an important role in translation. These data provide evidence that under higher oxygen tension, aromatic amino acids activated BMMSCs signaling pathways that were more related to translation, protein synthesis and control of oxidative stress and cell survival.

Our western blot data under normoxic conditions showed an up-regulation of the MAPK/ERK pathway in BMMSCs treated with Trp alone or in combination with Tyr and Phe, which confirms the evidence that nutrient-activated BMMSCs signaling pathways are different between hypoxia and normoxia. These data suggest a role of Trp in stem cell survival, proliferation and differentiation via the MAPK pathway under higher oxygen tension.

Aromatic amino acids (particularly Tyr and Trp) have been shown to have potent anti-oxidant capacity at physiologically relevant concentrations [21]. This may be particularly relevant in an aging bone marrow microenvironment where there is increased generation of harmful reactive oxygen species.

We conclude that hypoxia up-regulates BMMSC proliferative signaling pathways, as low oxygen tension (3% O_2_) more closely resembles the normal physiologic conditions these cells are exposed to in their micro-niches and that the increase in oxygen tension that occurs with aging causes decreases in stem cell renewal capacity. Our data also suggest a role of aromatic amino acids in the control of signaling pathways related to oxidative stress, protein synthesis and cell survival under the higher oxygen levels/increased oxidative stress that occurs with aging.

## References

[1] Ding KH, Shi XM, Zhong Q, Kang B, Xie D, et al (2008) Impact of glucose-dependent insulinotropic peptide on age-induced bone loss. J Bone Miner Res 23: 536–543. 18072880 10.1359/JBMR.071202 PMC2669161

[2] Zhang W, Ou G, Hamrick M, Hill W, Borke J, et al (2008) Age-related changes in the osteogenic differentiation potential of mouse bone marrow stromal cells. J Bone Miner Res 23: 1118–1128. 18435580 10.1359/JBMR.080304 PMC2679384

[3] Bonjour JP (2005) Dietary protein: an essential nutrient for bone health. J Am Coll Nutr 24: 526S–536S. 16373952 10.1080/07315724.2005.10719501

[4] Wengreen HJ, Munger RG, West NA, Cutler DR, Corcoran CD, et al (2004) Dietary protein intake and risk of osteoporotic hip fracture in elderly residents of Utah. J Bone Miner Res 19: 537–545. 15005839 10.1359/JBMR.040208

[5] Conigrave AD, Quinn SJ, Brown EM (2000) L-amino acid sensing by the extracellular Ca2+-sensing receptor. Proc Natl Acad Sci U S A 97: 4814–4819. 10781086 10.1073/pnas.97.9.4814 PMC18315

[6] Kerstetter JE, Caseria DM, Mitnick ME, Ellison AF, Gay LF, et al (1997) Increased circulating concentrations of parathyroid hormone in healthy, young women consuming a protein-restricted diet. Am J Clin Nutr 66: 1188–1196. 9356538 10.1093/ajcn/66.5.1188

[7] Johnson NE, Alcantara EN, Linkswiler H (1970) Effect of level of protein intake on urinary and fecal calcium and calcium retention of young adult males. J Nutr 100: 1425–1430. 5481677 10.1093/jn/100.12.1425

[8] Allen LH, Oddoye EA, Margen S (1979) Protein-induced hypercalciuria: a longer term study. Am J Clin Nutr 32: 741–749. 433806 10.1093/ajcn/32.4.741

[9] Conboy IM, Conboy MJ, Wagers AJ, Girma ER, Weissman IL, et al (2005) Rejuvenation of aged progenitor cells by exposure to a young systemic environment. Nature 433: 760–764. 15716955 10.1038/nature03260

[10] Baksh D, Davies JE, Zandstra PW (2003) Adult human bone marrow-derived mesenchymal progenitor cells are capable of adhesion-independent survival and expansion. Exp Hematol 31: 723–732. 12901978 10.1016/s0301-472x(03)00106-1

[11] Ivanovic Z, Bartolozzi B, Bernabei PA, Cipolleschi MG, Rovida E, et al (2000) Incubation of murine bone marrow cells in hypoxia ensures the maintenance of marrow-repopulating ability together with the expansion of committed progenitors. Br J Haematol 108: 424–429. 10691876 10.1046/j.1365-2141.2000.01842.x

[12] Ivanovic Z, Dello Sbarba P, Trimoreau F, Faucher JL, Praloran V (2000) Primitive human HPCs are better maintained and expanded in vitro at 1 percent oxygen than at 20 percent. Transfusion 40: 1482–1488. 11134568 10.1046/j.1537-2995.2000.40121482.x

[13] Herberg S, Fulzele S, Yang N, Shi X, Hess M, et al (2013) Stromal cell-derived factor-1beta potentiates bone morphogenetic protein-2-stimulated osteoinduction of genetically engineered bone marrow-derived mesenchymal stem cells in vitro. Tissue Eng Part A 19: 1–13. 22779446 10.1089/ten.tea.2012.0085 PMC3530941

[14] Arounleut P, Bowser M, Upadhyay S, Shi XM, Fulzele S, et al (2013) Absence of Functional Leptin Receptor Isoforms in the POUND (Lepr(db/lb)) Mouse Is Associated with Muscle Atrophy and Altered Myoblast Proliferation and Differentiation. PLoS One 8: e72330. 23967295 10.1371/journal.pone.0072330 PMC3743798

[15] Conigrave AD, Franks AH, Brown EM, Quinn SJ (2002) L-amino acid sensing by the calcium-sensing receptor: a general mechanism for coupling protein and calcium metabolism? Eur J Clin Nutr 56: 1072–1080. 12428172 10.1038/sj.ejcn.1601463

[16] Conigrave AD, Mun HC, Lok HC (2007) Aromatic L-amino acids activate the calcium-sensing receptor. J Nutr 137: 1524S–1527S discussion 1548S. 17513419 10.1093/jn/137.6.1524S

[17] Hamrick MW, Ding KH, Pennington C, Chao YJ, Wu YD, et al (2006) Age-related loss of muscle mass and bone strength in mice is associated with a decline in physical activity and serum leptin. Bone 39: 845–853. 16750436 10.1016/j.bone.2006.04.011

[18] Mangi AA, Noiseux N, Kong D, He H, Rezvani M, et al (2003) Mesenchymal stem cells modified with Akt prevent remodeling and restore performance of infarcted hearts. Nat Med 9: 1195–1201. 12910262 10.1038/nm912

[19] Wang M, Zhang W, Crisostomo P, Markel T, Meldrum KK, et al (2007) STAT3 mediates bone marrow mesenchymal stem cell VEGF production. J Mol Cell Cardiol 42: 1009–1015. 17509611 10.1016/j.yjmcc.2007.04.010 PMC1993849

[20] Park HJ, Carr JR, Wang Z, Nogueira V, Hay N, et al (2009) FoxM1, a critical regulator of oxidative stress during oncogenesis. EMBO J 28: 2908–2918. 19696738 10.1038/emboj.2009.239 PMC2760115

[21] Meucci E, Mele M (1997) Amino acids and plasma antioxidant capacity. Amino Acids 12: 373–377.

